# Cardiac-induced motion of the pancreas and its effect on image quality of ultrahigh-resolution CT

**DOI:** 10.1186/s41747-023-00401-5

**Published:** 2024-01-04

**Authors:** Thomas Wesley Holmes, Zhou Yu, Richard Thompson, John N. Oshinski, Amir Pourmorteza

**Affiliations:** 1 Department of Radiology and Imaging Sciences, Emory University, 1364 Clifton Road NE, Atlanta, GA 30322 USA; 2 Canon Medical Research USA, Inc, 706 N. Deerpath Drive, Vernon Hills, IL 60061 USA; 3 Department of Biomedical Engineering, Emory University – Georgia Institute of Technology, 201 Dowman Drive, Atlanta, GA 30322 USA; 4 Winship Cancer Institute, Emory University, 1701 Uppergate Dr, Suite 5018A, Atlanta, GA 30322 USA

**Keywords:** Abdomen, Artifacts, Healthy volunteers, Magnetic resonance imaging, Tomography (x-ray computed)

## Abstract

Recent advancements in diagnostic CT detector technology have made it possible to resolve anatomical features smaller than 20 LP/cm, referred to as ultra-high-resolution (UHR) CT. Subtle biological motions that did not affect standard-resolution (SR) CT may not be neglected in UHR. This study aimed to quantify the cardiac-induced motion of the pancreas and simulate its impact on the image quality of UHR-CT. We measured the displacement of the head of the pancreas in three healthy volunteers using Displacement Encoding with Stimulated Echoes (DENSE) MRI. The results were used to simulate SR- and UHR-CT acquisitions affected by pancreatic motion.

We found pancreatic displacement in the 0.24–1.59 mm range during one cardiac cycle across the subjects. The greatest displacement was observed in the anterior–posterior direction. The time to peak displacement varied across subjects. Both SR and UHR images showed reduced image quality, as measured by radial modulation transfer function, due to cardiac-induced motion, but the motion artifacts caused more severe degradation in UHR acquisitions. Our investigation of cardiac-induced pancreatic displacement reveals its potential to degrade both standard and UHR-CT scans. To fully utilize the improvement in spatial resolution offered by UHR-CT, the effects of cardiac-induced motion in the abdomen need to be understood and corrected.

**Relevance statement **Advancements in CT detector technology have enhanced CT scanner spatial resolution to approximately 100 µm. Consequently, previously ignored biological motions such as the cardiac-induced motion of the pancreas now demand attention to fully utilize this improved resolution.

**Graphical Abstract:**

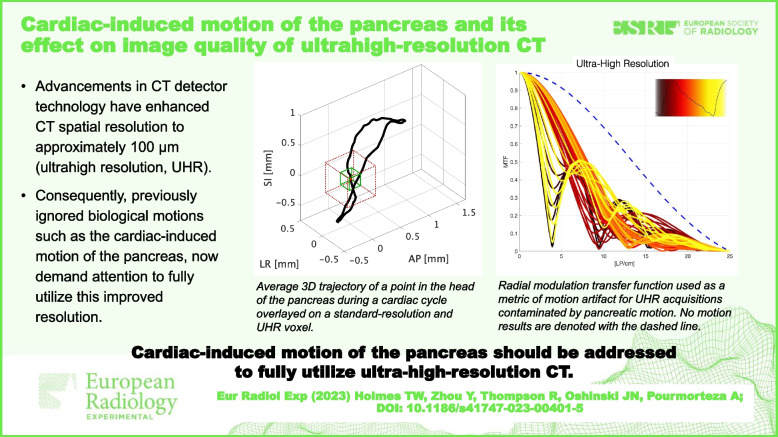

## Background

Recent advances in CT detectors have doubled the spatial resolution of CT scanners. This leap in commercial [1, 2] and prototype CT scanners [3–6] enables visualization of spatial frequencies larger than 20 LP/cm commonly referred to as ultra-high-resolution (UHR) CT. This technology is valuable in visualizing small anatomies and in detecting earlier changes in the body, *e.g.*, in response to treatments. Some examples include evaluating vasa vasorum of carotids [7] and improved visualization of small coronary arteries [8].

Many target anatomies in CT are in motion. The sources of these biological motions include voluntary movement of skeletal muscles, respiration, involuntary movement of flat muscles, *e.g.*, peristaltic motion of the gastrointestinal tract, and most importantly beating of the heart. In the timescales of a CT scan acquisition, which is usually less than 5 s and is done under breathholds, voluntary movements can be managed. Peristaltic motion is difficult to manage; however, its frequency is very low. The major source of biological motion affecting CT is therefore cardiac-induced. The motion of the heart affects the organs in the chest and abdomen, and the blood pressure wave that is carried by the aorta and major arteries throughout the body. There are many studies and commercial algorithms dedicated to the detection and compensation of the motion of the heart in standard-resolution (0.5-mm) cardiac CT [9], although it should be noted that most tissues undergo non-rigid deformation which is nearly impossible to correct completely. However, subtle cardiac-induced motions that did not affect the image quality in standard-resolution (SR) CT may not be ignored in UHR imaging of the heart and other organs.

In this work, we report MRI-based measurements of displacements in the pancreas as an example of a critical non-cardiac anatomy affected by cardiac-induced motion and simulated its effect on the image quality of SR- and UHR-CT.

## Methods

This pilot study consisted of 3 healthy volunteers, 2 males (27 and 28 years old) and 1 female (32 years old). For each subject, after acquiring localizer scans, we placed a point of interest (POI: 5 × 5 × 8 mm^3^) in the center of the head of the pancreas and acquired 2D displacement encoding with stimulated echoes (DENSE) MRIs in axial, sagittal, and coronal planes coinciding at the point of interest from 0% to ~ 90% of the R-R interval [10, 11]. This resulted in two independent measurements of the displacement of the POI in anterior–posterior (AP), left–right (LR), and superior-inferior (SI) directions. We used shape-preserving piecewise cubic Hermite interpolating polynomial (PCHIP) to estimate the trajectory for the remainder of the cardiac cycle. The average of the two measurements in each direction was used to infer the 3D trajectory of the POI during one cardiac cycle.

### MRI protocol

Two-dimensional DENSE scans were acquired on a 3-T MRI scanner (PrismaFit, Siemens Healthcare, Erlangen, Germany). Scans were initialized with a tagging pulse at the peak of the electrocardiogram signal, and a total of 49–61 frames were acquired over the cardiac cycle depending on the subject’s heart rate with the following parameters: flip angle = 15°, temporal resolution/frame = 16 ms per direction (62.5 frames per second), encoding frequency = 0.3 cycles/mm, field of view = 300 × 300 mm, reconstruction matrix = 256 × 256, pixel size = 1.17 × 1.17 mm, and slice thickness = 8 mm. Each acquisition was performed during a single breathhold with a duration of ~ 20 s. The accuracy of this DENSE-MRI protocol is reported to be between 15 and 50 μm [12]. The displacement information in DENSE-MRI is encoded in the phase signal of each pixel. It is important to note that DENSE-MRI does not track image features based on magnitude images over time. Instead, subpixel displacements result in phase shifts within that pixel, accrued during predefined time intervals (TR), and postprocessed to calculate the mean displacement within that pixel over TR. Consequently, the pixels can be larger than the measured motion. Therefore, while the resolution of the displacement map is dictated by pixel size, the accuracy of motion in each pixel is independent of its size.

### CT simulations

We devised an analytical model to isolate and investigate the effects of cardiac-induced motion on the image quality of a simulated CT scanner in SR and UHR acquisitions. The model consisted of a pulsating disk (diameter at rest = 30 mm, 400 HU), inside a water cylinder (diameter = 150 mm, 0 HU) both centered at the isocenter. The diameter of the disk pulsated at the same rate and amplitude as the anterior–posterior displacement measured in the subjects, with R-R length adjusted to 1 s for simplicity. The diameter at rest was selected to be in the middle of the range of the head of the pancreas in healthy adults measured by CT [13].

Fan-beam projections were calculated analytically for a single-row curved detector geometry using the Radon transform and were reconstructed with a filtered-backprojection algorithm with a resolution-preserving Shepp-Logan filter [14]. Scanner parameters included the following: source-to-detector distance = 2 m, source-to-isocenter distance = 1 m, 3,500 projections over rotation time = 350 ms, and standard-resolution and UHR detector pixel/image voxel sizes were 0.5 × 0.5/0.5 × 0.5 × 0.5 and 0.2 × 0.2/0.2 × 0.2 × 0.2 all in mm, respectively. We repeated the simulation using different starting times in the R-R interval for the 350-ms acquisition window every 20 ms to find optimal electrocardiogram triggering time to minimize motion artifact.

We measured the average radial modulation transfer function (rMTF) [15] of the entire edge of the disk as a metric of motion artifact. Optimal scan start time was defined as when the root mean square error (RMSE) in rMTF was minimized.

## Results

### DENSE-MRI measurements

**Fig. 1:**
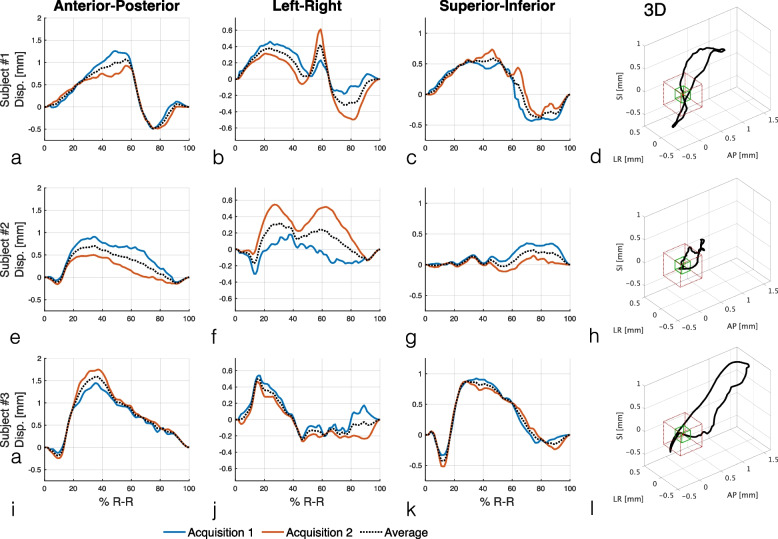
Displacements of a point-of-interest (POI) in the head of the pancreas of three healthy volunteers in anterior–posterior (**a**, **e**, **i**), left–right (**b**, **f**, **j**), and superior-inferior (**c**, **g**, **k**) principal directions and their average 3D trajectory (**d**, **h**, **l**). DENSE-MRIs were acquired during separate breathholds in the three anatomical planes (axial, sagittal, and coronal) coinciding at the POI, resulting in two independent measurements of displacement in each principal direction. The average 3D trajectory is overlayed on a standard-resolution voxel (red cube) and an ultra-high-resolution one (green cube)

Figure 1 shows the measured displacement of the POI in the center of the head of the pancreas in the three anatomical directions in all subjects. Maximum displacements, time to peak displacement, and optimal scan times were different for the three subjects (Table 1).

**Table 1:** The displacements are reported in the three major anatomical directions: anterior–posterior (A-P), left–right (L-R), and superior-inferior (S-I). Time to peak displacement and optimal scan time based on the radial MTF motion artifact metric are also reported

	Peak displacement (mm)			Time to peak (%R-R)			Optimal scan time (%R-R)	
Subject	A-P	L-R	S-I	A-P	L-R	S-I	SR	UHR
#1	1.07	0.42	0.59	56%	59%	46%	28%	84%
#2	0.70	0.32	0.24	34%	31%	74%	78%	78%
#3	1.59	0.50	0.88	36%	16%	29%	68%	60%

There was good agreement between the two independent measurements of displacement in subjects #1 and #3. Subject #2 showed larger variations, which may be attributed to different positions of the diaphragm at the start of breathholds.

Average 3D trajectory of the POI with respect to SR and UHR voxel sizes for the three subjects is shown in Fig. 1. The trajectory remained mostly within the bounds of a SR voxel for subject #2 while the other two cases showed displacements well beyond it.

### CT simulations

**Fig. 2:**
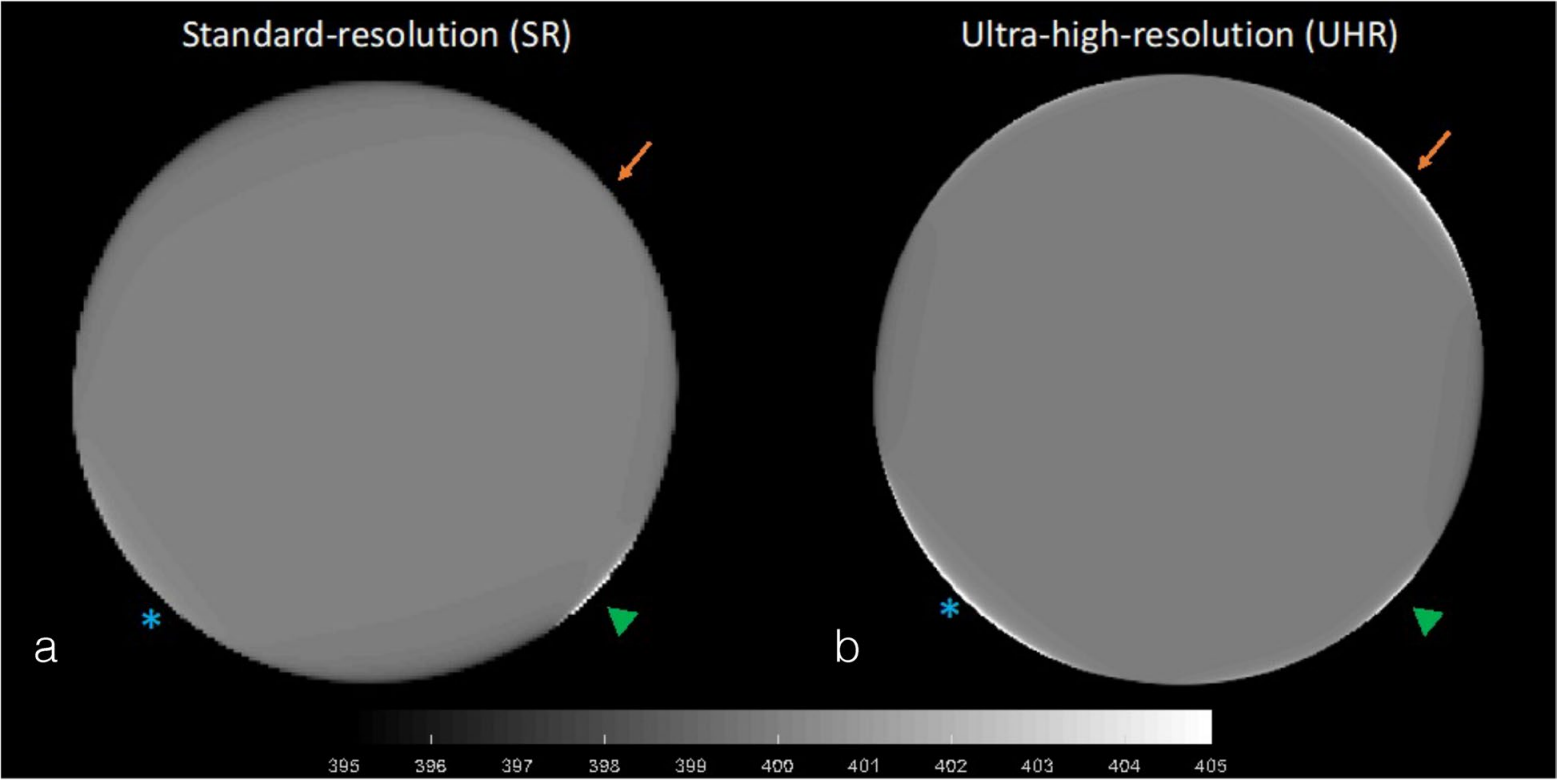
Sample CT images of a 3-cm diameter pulsating disk imaged at standard-resolution (**a**) and ultra-high-resolution (**b**), contaminated by pancreatic motion artifact. The diameter of the disk pulsated at the same rate and amplitude as the anterior–posterior displacement of the head of the pancreas measured by DENSE-MRI in subject #3. Cardiac-induced motion artifacts are more pronounced in the UHR image. The arrows, stars, and triangles mark some of the significant artifacts

Figure 2 presents an example of deterioration in image quality in SR- and UHR-CT due to cardiac-induced motion of the pancreas of subject #3. The artifacts were more pronounced and stronger in the UHR compared to the SR image.

### Effect of scan start time

**Fig. 3:**
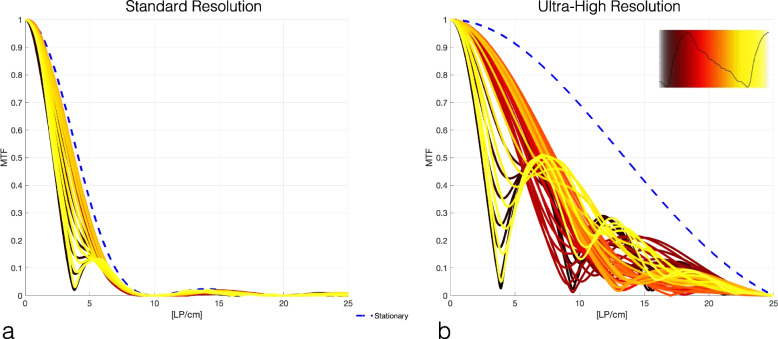
Standard resolution (**a**) and ultra-high resolution (**b**) radial modulation transfer function (MTF) of the 3-cm diameter pulsating disk as metric for degradation of image quality due to cardiac-induced motion of the pancreas for subject #3 (peak displacement = 1.59 mm). The dashed lines indicate the MTF of the disk at rest, and the colored lines denote the MTF of CT images at different scan start times during the R-R cycle. The inset depicts the anterior–posterior displacement of the subject (black line) and the colored rectangles denote different CT acquisition windows

Figure 3 shows the effect of scan start time on the severity of motion artifacts as measured by rMTF. The results indicate that both SR and UHR images were affected by motion artifacts. SR scans acquired at the right time did not present significant degrading, whereas all UHR scans, regardless of their start time, were affected by motion artifact. Optimal scan start time varied across the subjects and is also different for SR and UHR scan except in subject #2 (Table 1).

## Discussion

Our study of investigating the effects of cardiac-induced motion on a new imaging mode was inspired by Enzmann and Pelc [16] who reported displacements in the brain during a cardiac cycle. Recent brain DENSE-MRI studies report displacements of up to 369 μm and 148 μm in the brains of patients with Chiari malformation and normal subjects, respectively [17].

Our results indicate that the pancreatic displacement is large enough to cause degradation in both standard resolution and UHR-CT. The displacement waveforms were different across subjects with the largest displacements (> 0.8 mm) measured in the A-P direction. We postulate that the cardiac-induced motion of the pancreas has at least three components, in temporal order:

1. Mechanical motion of the heart and the diaphragm moving the pancreas mostly in S-I.
2. Pulsation of the aorta, which is located very close to the head and neck of the pancreas, moving it in the axial plane (A-P, L-R); this seems to be the largest component of the motion and matches time-to-peak displacements at 30% of R-R cycle in healthy subjects previously reported [18].
3. Systolic blood pressure wave causes the pancreas to bloom in all directions and seems to be the second largest component of the motion.

The relative delay across these components and the interplay of cardiac-induced motion of surrounding organs may depend on various physiological factors, especially those affecting blood pressure wave velocity, such as height, weight, blood pressure, and atherosclerosis. While we used the pancreas as an example in this study, a similar argument could be made for the characterization of hepatic and renal masses. A larger controlled study is warranted to investigate the details of cardiac-induced motion of the abdominal organs and to find optimal gating or motion correction solutions.

One way to reduce motion artifacts in CT is to speed up acquisition time; this may be achieved by using high helical pitch (> 1.5) scans; however, such scans are prone to geometric distortion [19, 20]. While tolerable in cardiac CT, distortions are especially detrimental in imaging the pancreas due to the small size of enhancing nodules or walls of cystic neoplasms that have a high chance of malignancy [21]. The tradeoff between motion artifacts and geometric distortion remains to be investigated.

This study has many limitations. Aside from the small sample size of the study, the MRIs were acquired during long breathholds and over multiple heartbeats. Therefore, the reported results are the “average effect” of cardiac-induced motion over many heartbeats and varied between the two independent measurements (especially in subject #2); most CT scans are acquired in one heartbeat and therefore may show more variability in displacements. Our simulated CT studies were performed in 2D for the sake of simplicity; we expect 3D simulations would show greater degradation of image quality [2]. Lastly, we chose a simple image quality metric (rMTF); more detailed studies with task-based detectability metrics will help assess diagnostic implications of cardiac-induced motion.

In conclusion, our findings underscore the importance of understanding and addressing the effects of cardiac-induced motion in the abdomen to optimize the advantages offered by UHR-CT.

## Data Availability

The datasets generated and/or analyzed during the current study are not publicly available due to active and ongoing research and grant writing by the authors on this subject. However, they are available from the corresponding author upon reasonable request.

## Abbreviations

CT: Computed tomography
DENSE: Displacement encoding with simulated echoes
MRI: Magnetic resonance imaging
MTF: Modulation transfer function
SR: Standard resolution
UHR: Ultra-high-resolution
